# Introduction

**DOI:** 10.1038/sj.bjc.6601628

**Published:** 2004-03-05

**Authors:** T Howell

**Affiliations:** 1 Cancer Research UK Department of Medical Oncology, Christie Hospital NHS Trust, Wilmslow Road, Withington, Manchester M20 4BX, UK

**Keywords:** antioestrogens, breast cancer, endocrine therapy, fulvestrant, ‘Faslodex’

Progestins, high-dose oestrogens, selective oestrogen receptor modulators (SERMs), aromatase inhibitors (AIs), and, most recently, the new type of oestrogen receptor (ER) antagonist fulvestrant (‘Faslodex’) have all been used for the treatment of hormone-sensitive advanced breast cancer in postmenopausal women. Tamoxifen was the ‘gold standard’ hormonal therapy for both pre- and postmenopausal women for many years. Recently, the third-generation nonsteroidal AIs anastrozole and letrozole and the steroidal AI exemestane have been shown to be more effective than tamoxifen as first-line treatment in postmenopausal women with hormone-sensitive advanced disease, leading to approval in this treatment setting. Different types of endocrine treatments are associated with different side effects, which may include nausea, vaginal bleeding and weight gain. SERMs such as tamoxifen have oestrogen agonist effects in a number of tissues including breast and uterus, which can cause ‘tumour flare’ and may increase the risk of endometrial cancer. Effects on lipid profile may increase the risk of thromboembolic events.

Most patients will eventually become resistant to endocrine agents. In an attempt to extend the time window in which well-tolerated treatments may be used, it is common clinical practice to use two or more different endocrine therapies in sequence. For this to be successful, it is necessary to use endocrine agents with different mechanisms of action that do not possess crossresistance with any hormonal therapies previously used. New endocrine therapies with novel mechanisms of action provide opportunities not only for optimising the efficacy of breast cancer treatment, but may also allow a longer time in which endocrine therapies can be used before reliance upon cytotoxic chemotherapy is necessary.

Fulvestrant is a new type of endocrine agent for the treatment of hormone-sensitive advanced breast cancer in postmenopausal women who have progressed on prior endocrine therapy. Unlike tamoxifen, fulvestrant has no oestrogen agonist activity. This supplement contains four papers that together provide a comprehensive review of fulvestrant. The first of these, by C Kent Osborne, Robert Nicholson and Alan Wakeling, provides an overview of the current understanding of ER signalling and its effect on breast cancer development. It details how fulvestrant acts to produce downregulation of cellular levels of the ER. Following this, John Robertson and Mike Harrison review the pharmacokinetics and metabolism of fulvestrant. In the third paper, John Robertson and Ignace Vergote report on the clinical data for fulvestrant, from the early efficacy and tolerability studies through to the three randomised phase III trials for fulvestrant that have been conducted in postmenopausal women with hormone-sensitive advanced breast cancer; two of these compared fulvestrant with anastrozole in patients who had progressed on prior endocrine therapy, and the third compared fulvestrant with tamoxifen as first-line endocrine treatment. Finally, Stephen Johnston reviews the evidence suggesting the versatility of fulvestrant in the sequencing of hormonal therapy, and considers the options for optimising the endocrine sequence cascade.

